# Correction: The β-cyclodextrin-modified nanosized ZSM-5 zeolite as a carrier for curcumin

**DOI:** 10.1039/d0ra90085k

**Published:** 2020-09-24

**Authors:** Niloofar Rahmani, Shahin Amani, Amir Bagheri Garmarudi, Mohammadreza Khanmohammadi

**Affiliations:** Department of Chemistry, Faculty of Science, Imam Khomeini International University Qazvin Iran

## Abstract

Correction for ‘The β-cyclodextrin-modified nanosized ZSM-5 zeolite as a carrier for curcumin’ by Shahin Amani *et al.*, *RSC Adv.*, 2019, **9**, 32348–32356, DOI: 10.1039/C9RA04739E.

The authors regret that the names of the authors were listed incorrectly in the original article. The corrected author list is as shown above.

The Royal Society of Chemistry apologises for these errors and any consequent inconvenience to authors and readers.

## Supplementary Material

